# Diagnostic Utility of Fractional Anisotropy and the Apparent Diffusion Coefficient in the Median Nerve: A Nerve Conduction Velocity-Stratified Evaluation Using 3T MRI

**DOI:** 10.7759/cureus.97029

**Published:** 2025-11-17

**Authors:** Tsutomu Inaoka, Rui Iwata, Minori Kasai, Kenta Ninohira, Takamitsu Uchi, Rumiko Ishikawa, Shusuke Kasuya, Osamu Takahashi, Ryosuke Sakai, Hiroyuki Nakazawa, Masahiko Kishi, Ryuji Sakakibara, Hitoshi Terada

**Affiliations:** 1 Department of Radiology, Toho University Sakura Medical Center, Sakura, JPN; 2 Department of Clinical Physiological Function Laboratory, Toho University Sakura Medical Center, Sakura, JPN; 3 Department of Central Radiology, Toho University Sakura Medical Center, Sakura, JPN; 4 Department of Neurology, Toho University Ohashi Medical Center, Meguro, JPN; 5 Department of Neurology, Neurology Clinic Tsudanuma, Chiba, JPN

**Keywords:** adc, dti, dwi, fa, median nerve, nerve conduction velocity, peripheral neuropathy

## Abstract

Objectives: This study aimed to evaluate the clinical utility of fractional anisotropy (FA) and the apparent diffusion coefficient (ADC) values obtained by diffusion tensor imaging (DTI) of the median nerve at the wrist, in human subjects stratified by nerve conduction velocity (NCV) findings.

Materials and methods: Wrist DTI was performed on a 3T scanner using a dedicated flex coil. A total of 85 wrists from 43 NCV-normal subjects and 17 wrists from 10 NCV-abnormal subjects were included. The normal subjects were asymptomatic, with no upper-extremity pathology. The abnormal subjects had suspected or subclinical median nerve impairment confirmed by abnormal NCV findings. FA and ADC values were extracted, and the statistical analyses included the correlation with age and sex (in NCV-normal subjects), group comparisons, and a receiver operating characteristic (ROC) analysis to determine the diagnostic utility of FA and the ADC.

Results: In the NCV-normal subjects, the mean FA was 0.535 ± 0.072, and the mean ADC was 0.756 ± 0.126 × 10^−3 ^mm^2^/s. FA showed a mild negative correlation with age (r = −0.35, p=0.001), and the ADC showed a mild positive correlation with age (r = 0.29, p=0.008). No significant effects of age group, sex, or their interaction were observed for either metric (p > 0.05). Compared to the NCV-normal group, the abnormal group exhibited significantly lower FA (p = 0.027) and significantly higher ADC (p < 0.001) values. The ROC analysis demonstrated higher diagnostic performance for the ADC (area under the curve (AUC): 0.774; Youden index: 0.443) (p < 0.001) than for FA (AUC: 0.640; Youden index: 0.282) (p = 0.065). Combining both FA and ADC metrics and using logistic regression did not improve the performance over ADC alone (AUC: 0.775; Youden index: 0.443).

Conclusion: FA and ADC values both reflected median nerve impairment confirmed by the NCV. The ADC showed a trend toward superior diagnostic performance in distinguishing NCV-abnormal cases compared to the FA. These findings suggest the potential value of ADC as a clinically relevant imaging biomarker for peripheral nerve evaluations.

## Introduction

Peripheral neuropathy, or peripheral nerve impairment, can arise from a variety of causes, including diabetes, trauma, autoimmune diseases, kidney dysfunction, and genetic disorders. The diagnosis of peripheral neuropathy typically relies on clinical symptoms, physical examinations, medical history, blood tests, and nerve conduction velocity (NCV) studies; however, these methods do not allow a direct assessment of peripheral nerves, lesion localization, or their underlying pathologies [1-3]. Although ultrasound, which provides real-time imaging of peripheral nerves and surrounding structures, is widely used in clinical practice, its diagnostic value is limited by operator dependency and constraints in both contrast and spatial resolution. Consequently, there is growing interest in the use of magnetic resonance imaging (MRI) for peripheral nerve evaluations [1,2].

In addition to conventional MRI, diffusion tensor imaging (DTI) has been used for the noninvasive visualization and quantification of the microstructural properties of peripheral nerves. Among the key DTI-derived metrics, fractional anisotropy (FA) reflects the directional coherence and structural integrity of nerve fibers, and the apparent diffusion coefficient (ADC) quantifies the overall diffusivity within tissues. Both FA and the ADC have been studied as potential imaging biomarkers for detecting peripheral nerve pathologies such as demyelination, axonal injury, and edema [1-6].

It has been demonstrated that DTI metrics reflect structural alterations in the median nerve, with FA values typically decreased and ADC values increased [1,3,6-13]. Although FA values are considered more sensitive for detecting median nerve impairment, a significant overlap in FA values between normal and abnormal subjects has been reported, along with regional variability in FA values depending on the anatomical location [9,14-17]. Since ADC values are derived from diffusion-weighted imaging (DWI), similar to DTI, they are more readily applicable in routine clinical practice and have shown potential in detecting pathological changes in peripheral nerves [1,3,4,18,19]. Accordingly, while the usefulness of FA in the evaluation of peripheral neuropathy has been well-documented, we hypothesized that ADC could also be valuable in routine clinical practice.

Therefore, we conducted the present study to determine the FA and ADC values obtained by DTI of the median nerve at the wrist with the use of 3T MRI in subjects classified as either "normal" or "abnormal" based on their NCV findings. We further investigated the influence of age and sex on these metrics in the NCV-normal subjects and assessed the diagnostic utility of FA and the ADC by comparing DTI findings between NCV-normal and NCV-abnormal subjects.

## Materials and methods

This study was approved by the ethics committee of the Toho University Sakura Medical Center (approval nos. S23039_S19067(2014-011), S19067, and 2014-011). Written informed consent for study participation was obtained from all of the normal subjects. Informed consent was waived by the ethics committee for patients with suspected median nerve impairment, and study information was disclosed to them with an opportunity to opt out.

MRI acquisition protocol for the wrist

MRI was performed on each wrist separately using a 3T scanner (Magnetom Skyra, Siemens Healthcare, Erlangen, Germany) with a dedicated flex coil. The imaging protocol was as follows: single-shot echo planar imaging in the axial plane; field of view, 130 mm; matrix, 100 × 100, repetition time (TR), 6500 ms; echo time (TE), 81-83 ms; distance factor, 0%-20%; bandwidth, 1220 Hz/pixel; the parallel imaging technique (PAT), GeneRalized Autocalibrating Partially Parallel Acquisitions (GRAPPA); fat suppression, SPectral Attenuated Inversion Recovery (SPAIR); slice thickness, 3-4 mm; b-values, 0 and 1400 s/mm²; motion probing gradient (MPG) directions, 10; and number of excitations (NEX), 1-3.

NCV-normal subjects

**Figure 1:**
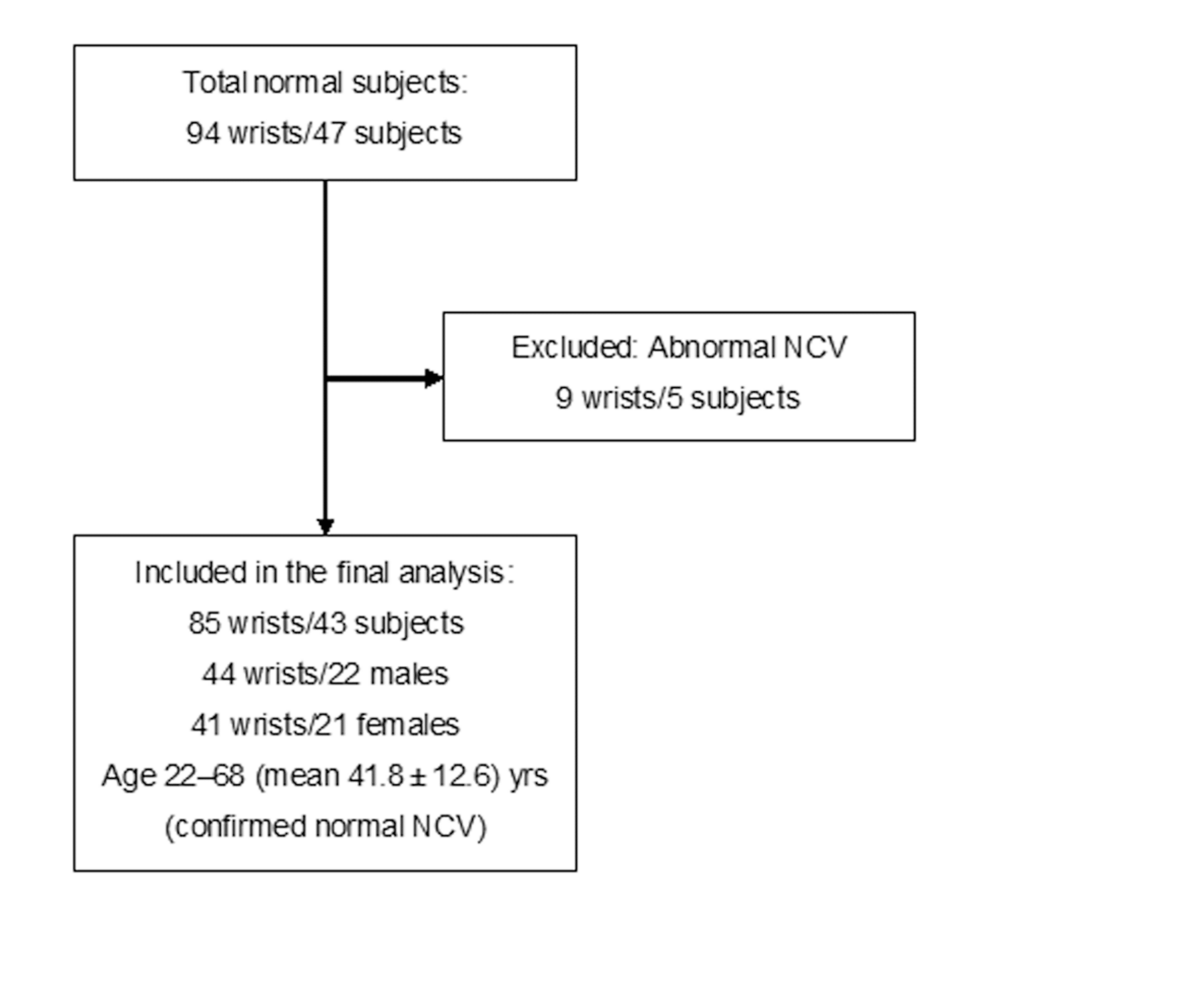
The subject selection flowcharts for the NCV-normal subject study NCV: nerve conduction velocity All subjects underwent bilateral wrist MRI. Because one subject had an abnormal NCV in one wrist and a normal NCV in the other, this subject was effectively counted twice; however, the total number of wrists remains consistent

A total of 94 wrists from 47 normal subjects underwent wrist MRI for the evaluation of the median nerve between January 2015 and June 2016. All subjects underwent bilateral wrist MRI. The inclusion criteria for the normal subjects were as follows: age ≥20 years, no history of hand or wrist injury, disease, or surgery; no self-reported symptoms of significant numbness or motor dysfunction; and the absence of standard contraindications for an MRI examination. All of the study subjects underwent an NCV study, which was conducted by certified technologists. NCV studies of the median nerve were evaluated using our institutional reference values, which are consistent with the standard clinical criteria in Japan. The MRI data for nine wrists of five subjects were excluded because of abnormal NCV results: the two wrists of one subject showed decreased motor and sensory NCVs, and seven wrists of four subjects showed decreased sensory NCVs. As a result, 85 wrists from 43 subjects were included in the final analysis as normal subjects (age range 22-68 years, mean age 41.8 ± 12.6 years), comprising 44 wrists from 22 males (age range 23-68 years, mean age 42.9 ± 13.8 years) and 41 wrists from 21 females (age range 22-61 years, mean age 40.7 ± 11.4 years). The NCV-normal subject selection is illustrated in Figure 1.

NCV-abnormal subjects

Eight patients were clinically suspected of having median nerve impairment by the internal medicine department of our hospital, and they underwent both an NCV examination and wrist MRI between January 2015 and February 2024. Among them, each patient had rheumatoid arthritis, diabetes mellitus, or both diabetes mellitus and myasthenia gravis as underlying conditions. Two patients were diagnosed with carpal tunnel syndrome. Eight wrists of five patients showed abnormal NCV results and were included in the abnormal group, and the data of five wrists with normal NCV among the other three patients were excluded.

**Figure 2:**
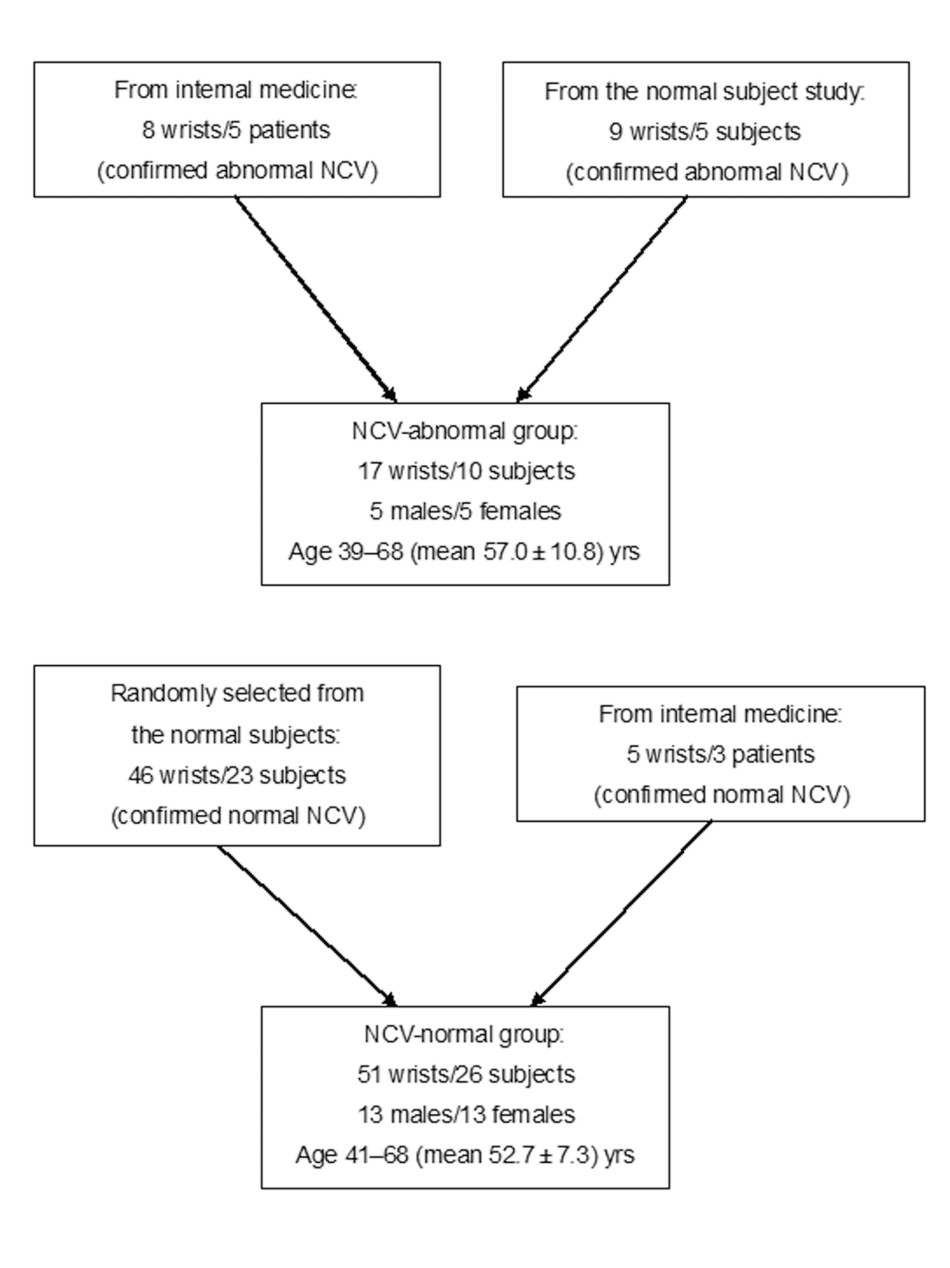
The subject selection flowcharts for the comparisons of the NCV-normal and -abnormal groups NCV: nerve conduction velocity We included the subclinical subjects from the NCV-normal subject study in the NCV-abnormal group since we believed that doing so would better reflect the distribution encountered in real-world clinical settings

In addition, the nine wrists of five subjects identified as having abnormal NCV during the normal-subject screening phase of this study described above were included in the abnormal group. Of these five subjects, one had undergone chemotherapy, one had cervical disc herniation, and three had no identifiable cause of the abnormality. We included these subclinical subjects since we believed that doing so would better reflect the distribution encountered in real-world clinical settings. As a result, 17 wrists from 10 subjects were assigned to the abnormal group (five males and five females; age range 39-69 years, mean 57.0 ± 10.8 years). The selection of the NCV-abnormal group subjects is illustrated in Figure 2.

Image analysis

FA and ADC values were obtained by manually placing a polygonal region of interest (ROI) that encompassed the entire cross-sectional area of the median nerve at the carpal tunnel level on wrist MRI. Measurements were performed on axial images between the proximal margin of the pisiform and the distal end of the radius, as described [10,12,13,20,21]. The locations were confirmed by using the anatomic location on 3D T1-weighted gradient-echo coronal and T2-weighted fast spin-echo axial images. The mean ROI size was 16.1 mm^2^ for the FA measurements and 17.0 mm^2^ for the ADC measurements. All ROI measurements for both FA and ADC were performed in a blinded manner on a single slice.

Statistical analysis

We determined the overall correlations among the FA values, ADC values, and age in the NCV-normal subjects and then categorized these subjects into five age groups: 20-29, 30-39, 40-49, 50-59, and ≥60 years old. Pearson's correlation tests were used to assess the relationships between FA values, ADC values, and age. The FA and ADC values were compared among the age groups and between the sexes by a two-way analysis of variance (ANOVA).

For the comparison of the NCV-normal group's FA and ADC values with those of the NCV-abnormal group, in addition to the five wrists of three patients with normal NCV, we randomly selected 46 wrists of 23 normal subjects to match the age and sex distribution of the abnormal group. Consequently, the normal group comprised 51 wrists of 26 subjects (13 males and 13 females; age range 41-68 years, mean 52.7 ± 7.3 years). The χ^2^-test was used to examine the sex distribution, and Student's t-test was used to compare ages between groups.

The FA and ADC values were compared between the normal and abnormal groups by Student's t-test, and receiver operating characteristic (ROC) analyses were then performed for both the FA and ADC values, with cutoff values determined based on the Youden index. Statistical analyses were conducted using IBM SPSS Statistics for Windows, Version 28 (Released 2021; IBM Corp., Armonk, New York, United States). Probability (p)-values <0.05 were considered significant.

## Results

NCV-normal subjects

In the NCV-normal group, the mean FA value was 0.535 ± 0.072, and the mean ADC was 0.756 ± 0.126 (×10^−3^mm^2^/s). A mild negative correlation was observed between age and FA values in this group (r = −0.35, p = 0.001), and a mild positive correlation was found between age and the ADC values (r = 0.29, p = 0.008). Table 1 provides the FA and ADC values across the five age groups of NCV-normal subjects.

**Table 1:** The FA and ADC values by age group and sex in the NCV-normal subjects FA: fractional anisotropy; ADC: apparent diffusion coefficient; NCV: nerve conduction velocity The FA and ADC data are mean ± standard deviation. The NCV-normal group was defined as the subjects with normal NCV findings

Age group	Sex	Number of wrists	FA	ADC, ×10^−3 ^mm^2^/s
20-29	M	10	0.544 ± 0.082	0.691 ± 0.102
	F	10	0.570 ± 0.064	0.728 ± 0.135
30-39	M	10	0.539 ± 0.061	0.745 ± 0.106
	F	9	0.583 ± 0.077	0.740 ± 0.064
40-49	M	8	0.556 ± 0.082	0.656 ± 0.084
	F	10	0.510 ± 0.082	0.780 ± 0.155
50-59	M	10	0.528 ± 0.065	0.789 ± 0.122
	F	10	0.499 ± 0.042	0.842 ± 0.156
≥60	M	6	0.478 ± 0.058	0.816 ± 0.114
	F	2	0.500 ± 0.052	0.742 ± 0.103

The two-way ANOVA revealed no significant effects of the age group, sex, or their interaction on either the FA or ADC values (p > 0.05) (Table 2).

**Table 2:** Effects of the age group and sex on the FA and ADC values (two-way ANOVA) FA: fractional anisotropy; ADC: apparent diffusion coefficient; ANOVA: analysis of variance The two-way ANOVA revealed no significant effects of age group, sex, or their interaction on either the FA or ADC values (p > 0.05)

Measure	Factor	F-value	p-value
FA	Age group (5 groups)	2.315	0.065
	Sex (male/female)	0.05	0.823
	Age group × sex	1.422	0.235
ADC	Age group (5 groups)	2.299	0.067
	Sex (male/female)	1.108	0.296
	Age group × sex	1.343	0.262

NCV-normal and NCV-abnormal groups

The normal and abnormal groups were comparable in age (p = 0.179) and sex (p = 1.000), with no significant differences (p > 0.05). The mean FA and ADC values for the NCV-normal and NCV-abnormal groups are presented in Table 3. Significant between-group differences were observed in both the FA values (p = 0.027) and ADC values (p < 0.001).

**Table 3:** The FA and ADC values in the NCV-normal and NCV-abnormal groups FA: fractional anisotropy; ADC: apparent diffusion coefficient; NCV: nerve conduction velocity The FA and ADC data are mean ± standard deviation. Normal: subjects with normal NCV findings. Abnormal: subjects with abnormal NCV findings. Student's t-test revealed significant between-group differences in both the FA values and ADC values (p < 0.05)

Group	Number of wrists	FA	t-value	p-value	ADC, ×10^−3 ^mm^2^/s	t-value	p-value
Normal	51	0.514 ± 0.068	2.263	0.027	0.795 ± 0.143	-4.752	<0.001
Abnormal	17	0.469 ± 0.059			1.021 ± 0.233		

According to the ROC analysis, the areas under the curve (AUCs) were 0.640 (95% confidence interval (CI): 0.491-0.788) for the FA values (p = 0.065) and 0.774 (95% CI: 0.644-0.904) for the ADC values (p < 0.001). The cutoff value for FA was 0.495, yielding 0.73 sensitivity, 0.549 specificity, and 0.282 as the Youden index. For the ADC, the cutoff value was 0.927, with 0.60 sensitivity, 0.843 specificity, and a Youden index of 0.443. However, the pairwise comparison of AUCs revealed no significant difference (p = 0.115). A combined analysis using both FA and ADC values via logistic regression was also performed; however, the diagnostic performance (based on the AUC and Youden index) was nearly identical to that of the ADC alone (Table 4).

**Table 4:** Diagnostic performance of FA, ADC, and their combination for identifying NCV-abnormal cases FA: fractional anisotropy; ADC: apparent diffusion coefficient; NCV: nerve conduction velocity The ADC demonstrated a trend toward superior diagnostic performance in distinguishing NCV-abnormal cases compared with the FA, although the difference did not reach statistical significance

	AUC (95% CI)	p-value	Cutoff value	Sensitivity	Specificity	Youden index	Comparison p-value
FA	0.640 (0.491-0.788)	0.065	0.495	0.73	0.55	0.282	
ADC	0.774 (0.644-0.904)	<0.001	0.927 (×10^−3 ^mm^2^/s)	0.6	0.84	0.443	0.115 (vs FA)
FA + ADC	0.775 (0.646-0.904)		Not applicable	0.6	0.84	0.443	

**Figure 3:**
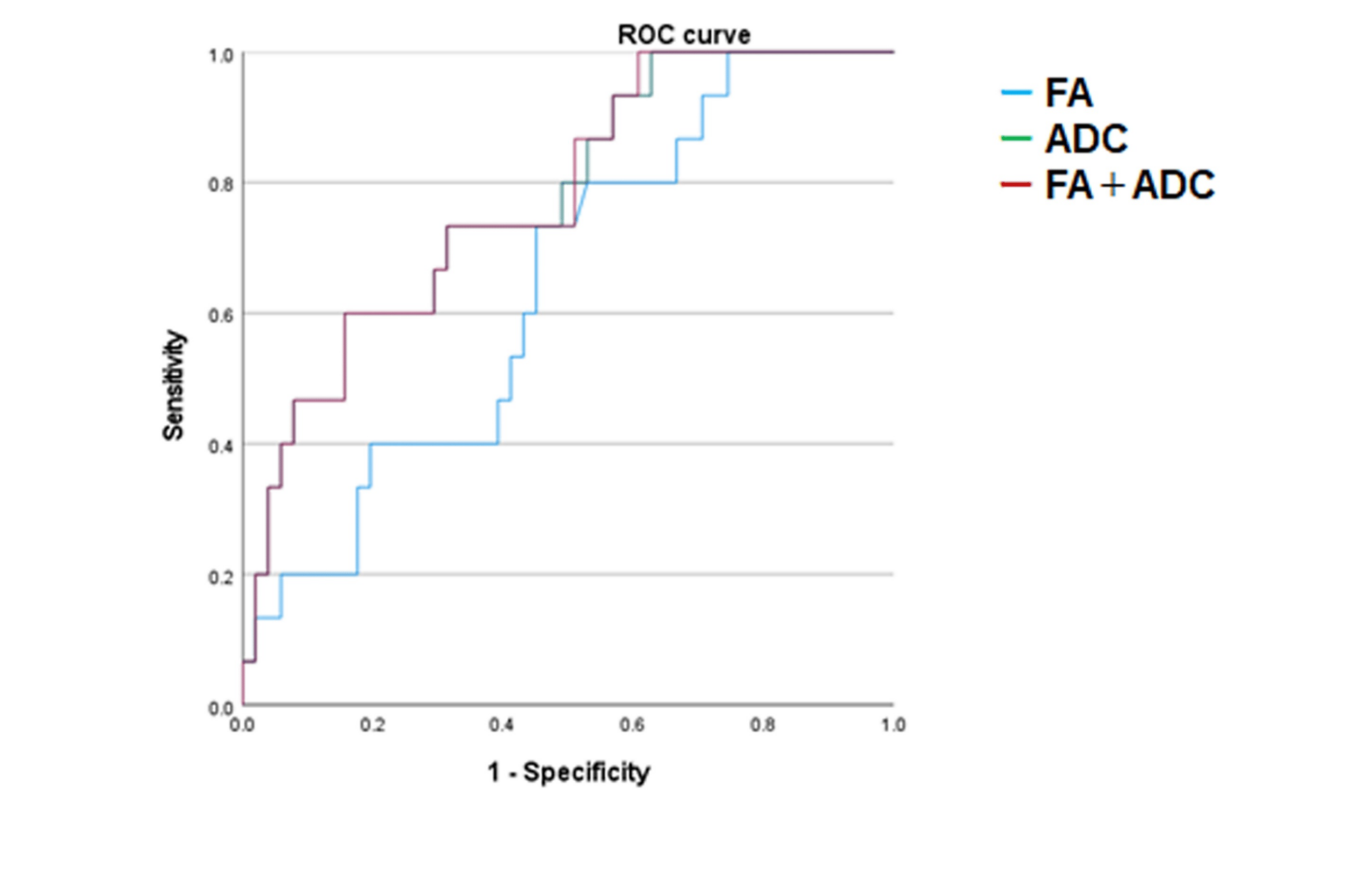
ROC curves of FA and ADC ROC: receiver operating characteristic; FA: fractional anisotropy; ADC: apparent diffusion coefficient; NCV: nerve conduction velocity; right blue line: FA; green line: ADC; red line: combined FA and ADC The ADC demonstrated a trend toward superior diagnostic performance in distinguishing NCV-abnormal cases compared with the FA. The combination of FA and ADC values did not outperform ADC alone

The ROC curves are presented in Figure 3.

**Figure 4:**
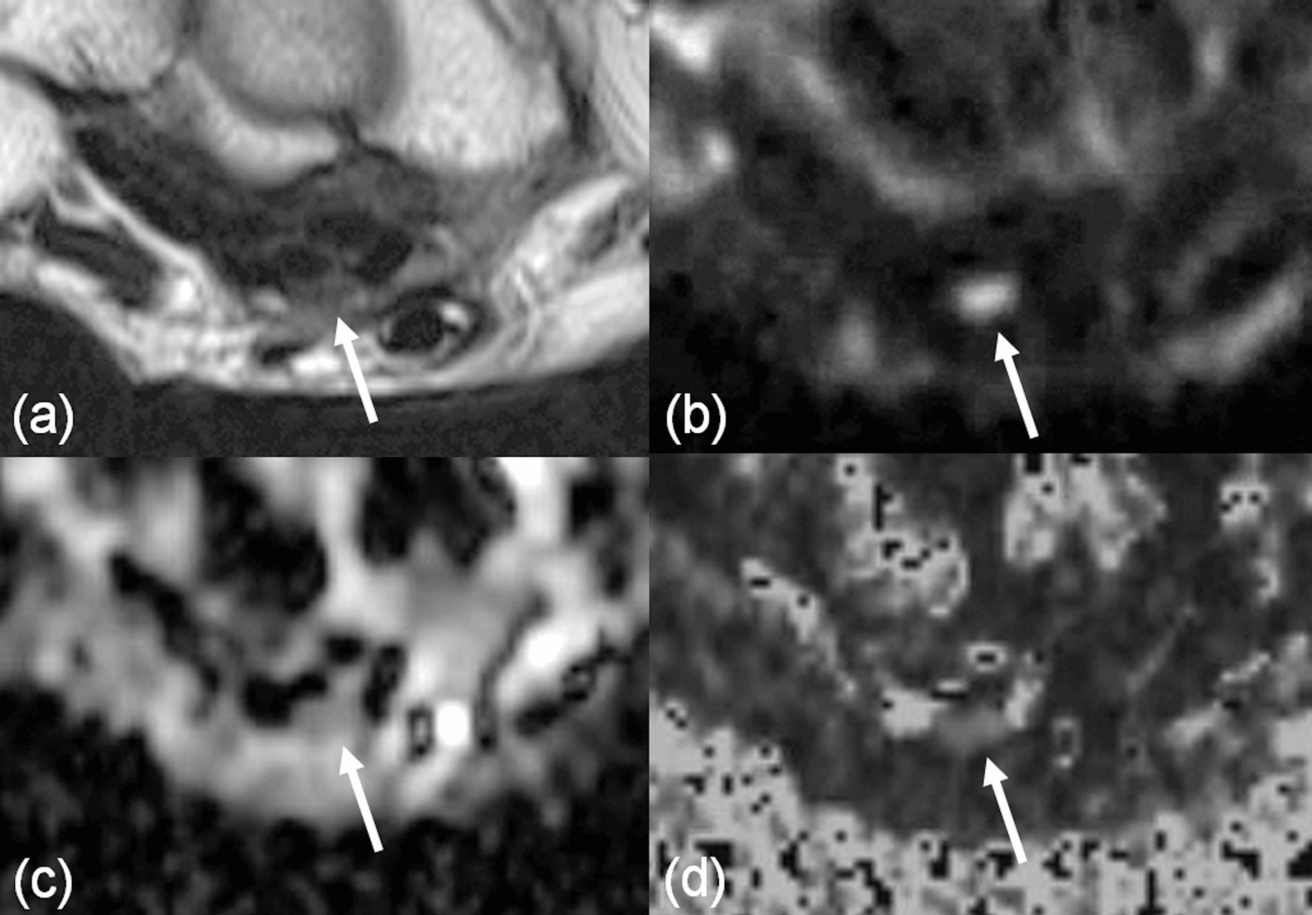
Diffusion tensor imaging of the wrist (NCV-normal case) FA: fractional anisotropy; ADC: apparent diffusion coefficient; NCV: nerve conduction velocity; T2WI: T2-weighted imaging; DWI: diffusion-weighted imaging A 68-year-old male patient presented with numbness in the fingertips of the left hand and was initially suspected of having carpal tunnel syndrome, but NCV was normal. Axial images of the left wrist at the level proximal to the pisiform bone are shown: (a) T2WI (4-mm thickness), (b) DWI (b = 1400, 4-mm thickness), (c) ADC map, and (d) FA map. The arrow indicates the median nerve. The ADC was 0.739 × 10⁻³ mm²/s, and the FA was 0.480

**Figure 5:**
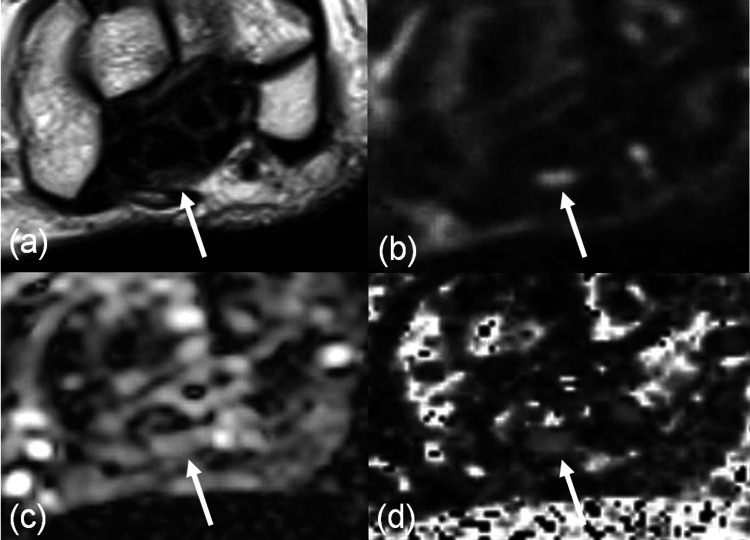
Diffusion tensor imaging of the wrist (NCV-abnormal case) FA: fractional anisotropy; ADC: apparent diffusion coefficient; NCV: nerve conduction velocity; T2WI: T2-weighted imaging; DWI: diffusion-weighted imaging A 69-year-old female patient presented with numbness in the right hand. NCV was abnormal, and the patient was ultimately diagnosed with carpal tunnel syndrome. Axial images of the right wrist at the level proximal to the pisiform bone are shown: (a) T2WI (4-mm thickness), (b) DWI (b = 1400, 4-mm thickness), (c) ADC map, and (d) FA map. The arrow indicates the median nerve. The ADC was 1.119 × 10⁻³ mm²/s, and the FA was 0.415

Representative cases are presented in Figures 4-5.

## Discussion

We evaluated DTI metrics, i.e., FA and the ADC, of the median nerve at the wrist, focusing on the impacts of age and sex in normal subjects screened by an NCV study, and we evaluated the clinical utility of these two parameters in subjects with abnormal NCV. According to previous reports [15,22], FA and ADC values are generally affected by age, but sex has little to no influence. With aging, a disorganization of nerve fibers' alignment and structure may lead to decreased FA and an increased ADC [22]. In a meta-analysis by Wang et al. [11], neither age nor sex showed any significant association with FA or ADC values. A more recent meta-analysis by Rojoa et al. [21] describes a negative association between age and FA values and a positive association between age and ADC values. In the present study, all of the subjects were stratified by an NCV study. To our knowledge, there have been few evaluations of normal subjects used as NCV controls. We observed mild correlations between age and both the FA and ADC values of the present NCV-normal subjects, but the two-way ANOVA revealed no significant differences across age groups or between the sexes among the participants. These results clearly suggest that age and sex are likely to have only a limited influence on DTI parameters of the median nerve, supporting a broader applicability of the DTI parameters across demographic subgroups in clinical practice.

Compared to the NCV-normal group, the NCV-abnormal group's FA values were significantly lower and their ADC values significantly higher, and the ADC values demonstrated a trend toward superior diagnostic performance in distinguishing NCV-abnormal cases compared with the FA values. Although the mean FA values differed significantly between the groups (t-test), the ROC analysis did not show a statistically significant difference in diagnostic performance. A combined logistic regression model incorporating both the FA and ADC data was performed, and we observed that it did not improve the diagnostic accuracy beyond that of ADC alone. Our results suggest that even the ADC alone may serve as a practical imaging biomarker for detecting neuropathic changes in the median nerve.

It has been reported that FA and ADC values reflect underlying structural alterations such as nerve fiber disorganization, demyelination, or axonal injury [7,8,10,11,20,23]. It has also been stated that FA values may be more sensitive for detecting mild-to-moderate carpal tunnel syndrome, but ADC values offer diagnostic performance that is comparable to that of FA values [12]. High variability in FA values across nerves and greater consistency in the ADC have been described [14]. As a possible reason for the trend toward ADC's superiority over FA for diagnosing median nerve abnormalities (as observed in the ROC analysis of the present study), we note that FA values have a limited dynamic range (0-1), which can result in overlapping values between NCV-normal and NCV-abnormal cases. In contrast, the ADC values in this study showed a relatively clearer separation, with a cutoff value at 0.923 × 10^⁻3 ^mm^2^/s.

DWI is widely used in clinical settings, and the ADC values obtained by DWI and DTI have been reported to be comparable [5]. An inverse relationship exists between the b-value and the ADC, in which increments of 100 mm^2^/s reduced the mean ADC by 0.04 × 10^-3 ^mm^2^/s [21], with lower b-values (e.g., 400-600 s/mm^2^) yielding higher ADC values [24]. Although the optimal b-value in DWI remains a matter of debate, values around 1000 s/mm^2^ are commonly used [21]. ADC values may vary slightly between vendors (as may FA values), but such differences are usually within the range of standard deviation (ADC: 0.05-0.06 × 10^−3 ^mm^2^/s; FA: 0.05-0.07) [16]. Although FA values differ by nerve type and location, ADC values remain relatively consistent [15]. An ADC is independent of the magnetic field strength [15]. Although FA reflects nerve anisotropy and integrity and is influenced by axonal and myelin properties, an ADC reflects the diffusion restriction of the molecules due to structural barriers such as cell membranes and myelin, and it increases with inflammation, edema, or injury [6]. Thus, although the ADC has low specificity, it is sensitive to pathological processes, and the ADC derived from DWI is more practical for evaluating both symptomatic and subclinical median nerve impairment in clinical practice [14,15].

Reported cutoff values for the ADC range from 1.00 to 1.31 × 10^−3 ^mm^2^/s, and the reported cutoff values for FA range from 0.44 to 0.55 [11]. In our present analyses, the threshold ADC value tended to be slightly lower than the reported values, while the threshold FA value was approximately in the mid-range of the reported values. This may be due to the stricter evaluation enabled by NCV-based screening, which may help to better discriminate subclinical median nerve impairment. Based on our findings, an ADC threshold of approx. 0.90 × 10^−3 ^mm^2^/s appears to be appropriate for detecting subclinical cases in routine practice.

There are several limitations to this study. The sample size, particularly in the NCV-abnormal group and certain age-sex subgroups, was relatively small, which may have limited the statistical power and affected the generalizability of the findings. In addition, both wrists from individual subjects were analyzed in most cases; therefore, the data points cannot be considered fully independent. Future research with larger cohorts is recommended. The study's cross-sectional design precludes any assessment of causal relationships or longitudinal changes in DTI metrics over time. Furthermore, although NCV abnormalities served as a clinical reference, a histopathological confirmation of nerve integrity was not available, which may limit the precision of the DTI-pathology correlation. Finally, in this study, FA and the ADC values were obtained from a single measurement, which represents a limitation with respect to inter- and intrarater reliability.

## Conclusions

The results of this DTI-based study demonstrated that FA and ADC values of the median nerve were minimally influenced by age and sex when subjects were screened based on NCV. Both the FA values and the ADC values differed significantly between the NCV-normal and NCV-abnormal groups. Our results suggest that ADC may be a useful noninvasive imaging biomarker for peripheral neuropathy, as it showed a trend toward greater effectiveness than FA in distinguishing NCV-abnormal from NCV-normal subjects. Although FA should not be disregarded, the technical limitations of DTI acquisition indicate that ADC derived from DWI may serve as a more practical tool in clinical settings. Although our findings require validation in larger, independent cohorts, they may contribute to improving diagnostic accuracy in routine clinical practice.

## References

[1] Chhabra A, Madhuranthakam AJ, Andreisek G (2018). Magnetic resonance neurography: current perspectives and literature review. Eur Radiol.

[2] Jeon T, Fung MM, Koch KM, Tan ET, Sneag DB (2018). Peripheral nerve diffusion tensor imaging: overview, pitfalls, and future directions. J Magn Reson Imaging.

[3] Hlis R, Poh F, Xi Y, Chhabra A (2019). Diffusion tensor imaging of diabetic amyotrophy. Skeletal Radiol.

[4] Tagliafico A, Calabrese M, Puntoni M, Pace D, Baio G, Neumaier CE, Martinoli C (2011). Brachial plexus MR imaging: accuracy and reproducibility of DTI-derived measurements and fibre tractography at 3.0-T. Eur Radiol.

[5] Chhabra A, Thakkar RS, Andreisek G (2013). Anatomic MR imaging and functional diffusion tensor imaging of peripheral nerve tumors and tumorlike conditions. AJNR Am J Neuroradiol.

[6] Wu C, Wang G, Zhao Y (2017). Assessment of tibial and common peroneal nerves in diabetic peripheral neuropathy by diffusion tensor imaging: a case control study. Eur Radiol.

[7] Stein D, Neufeld A, Pasternak O (2009). Diffusion tensor imaging of the median nerve in healthy and carpal tunnel syndrome subjects. J Magn Reson Imaging.

[8] Wang CK, Jou IM, Huang HW, Chen PY, Tsai HM, Liu YS, Lin CC (2012). Carpal tunnel syndrome assessed with diffusion tensor imaging: comparison with electrophysiological studies of patients and healthy volunteers. Eur J Radiol.

[9] Barcelo C, Faruch M, Lapègue F, Bayol MA, Sans N (2013). 3-T MRI with diffusion tensor imaging and tractography of the median nerve. Eur Radiol.

[10] Kwon BC, Koh SH, Hwang SY (2015). Optimal parameters and location for diffusion-tensor imaging in the diagnosis of carpal tunnel syndrome: a prospective matched case-control study. AJR Am J Roentgenol.

[11] Wang H, Ma J, Zhao L, Wang Y, Jia X (2016). Utility of MRI diffusion tensor imaging in carpal tunnel syndrome: a meta-analysis. Med Sci Monit.

[12] Razek AA, Shabana AA, El Saied TO, Alrefey N (2017). Diffusion tensor imaging of mild-moderate carpal tunnel syndrome: correlation with nerve conduction study and clinical tests. Clin Rheumatol.

[13] Liu C, Li HW, Wang L (2018). Optimal parameters and location for diffusion tensor imaging in the diagnosis of carpal tunnel syndrome: a meta-analysis. Clin Radiol.

[14] Hiltunen J, Suortti T, Arvela S, Seppä M, Joensuu R, Hari R (2005). Diffusion tensor imaging and tractography of distal peripheral nerves at 3 T. Clin Neurophysiol.

[15] Kabakci N, Gürses B, Firat Z, Bayram A, Uluğ AM, Kovanlikaya A, Kovanlikaya I (2007). Diffusion tensor imaging and tractography of median nerve: normative diffusion values. AJR Am J Roentgenol.

[16] Guggenberger R, Markovic D, Eppenberger P (2012). Assessment of median nerve with MR neurography by using diffusion-tensor imaging: normative and pathologic diffusion values. Radiology.

[17] Klauser AS, Abd Ellah M, Kremser C (2018). Carpal tunnel syndrome assessment with diffusion tensor imaging: value of fractional anisotropy and apparent diffusion coefficient. Eur Radiol.

[18] Amaya J, Lue B, Silva FD, Raspovic K, Xi Y, Chhabra A (2023). Diffusion-weighted MR imaging and utility of ADC measurements in characterizing nerve and muscle changes in diabetic patients on ankle DWI studies: a cross-sectional study. Eur Radiol.

[19] Evans AG, Morgan MD, Aiken BA (2023). Can diffusion tensor imaging apparent diffusion coefficient diagnose carpal tunnel syndrome? A systematic review and meta-analysis. Hand (N Y).

[20] Brienza M, Pujia F, Colaiacomo MC (2014). 3T diffusion tensor imaging and electroneurography of peripheral nerve: a morphofunctional analysis in carpal tunnel syndrome. J Neuroradiol.

[21] Rojoa D, Raheman F, Rassam J, Wade RG (2021). Meta-analysis of the normal diffusion tensor imaging values of the median nerve and how they change in carpal tunnel syndrome. Sci Rep.

[22] Kronlage M, Schwehr V, Schwarz D (2018). Peripheral nerve diffusion tensor imaging (DTI): normal values and demographic determinants in a cohort of 60 healthy individuals. Eur Radiol.

[23] Bulut HT, Yildirim A, Ekmekci B, Gunbey HP (2014). The diagnostic and grading value of diffusion tensor imaging in patients with carpal tunnel syndrome. Acad Radiol.

[24] Yao L, Gai N (2009). Median nerve cross-sectional area and MRI diffusion characteristics: normative values at the carpal tunnel. Skeletal Radiol.

